# Self-medication in Primary Dysmenorrhea among Medical and Nursing Undergraduate Students of a Tertiary Care Hospital: A Descriptive Cross-sectional Study

**DOI:** 10.31729/jnma.6397

**Published:** 2021-06-30

**Authors:** Jyoti Prabha Bharati, Sanjay Ulak, Merina Vaidya Shrestha, Sanjaya Mani Dixit, Anna Acharya, Ashish Bhattarai

**Affiliations:** 1 Department of Pharmacology, Kathmandu Medical College Teaching Hospital, Duwakot, Bhaktapur, Nepal; 2 Department of Paediatrics, Bhaktapur Hospital, Bhaktapur, Nepal; 3 Department of Community medicine, Kathmandu Medical College Teaching Hospital, Duwakot, Bhaktapur, Nepal.

**Keywords:** *awareness*, *dysmenorrhea*, *menstrual cycle*, *self-medications*

## Abstract

**Introduction:**

Primary dysmenorrhea is one of the most common gynecological problems among adolescent females. It is defined as painful menses in women with normal pelvic anatomy, usually beginning during adolescence. This study aims to find out prevalence of self-medication practice in primary dysmenorrhea among medical and nursing undergraduate students.

**Methods:**

A descriptive cross-sectional study was conducted in a tertiary care hospital from November 2020 to March 2021 after taking ethical approval from the Institutional Review Committee. Convenient sampling technique was used. A total of 269 female medical and nursing students with complaints of dysmenorrhea were enrolled and the remedial methods used by them such as self-medication, medical advice and home remedies for dysmenorrhea were asked using an online questionnaire delivered to participants. Data analysis was done in the Statistical Package of Social Sciences. Point estimate at 95% Confidence Interval was calculated along with frequency and proportion for binary data.

**Results:**

Self-medication practice for dysmenorrhea was reported in 175 (65%) of students. The prevalence of mild or moderate pain was commonly present in age group 21-25 years. commonly used for self-medication was mefenamic acid 121 (48%), followed by ibuprofen 51 (20.3%) and paracetamol 41 (16.3%).

**Conclusions:**

Self-medication practice among medical and nursing undergraduate students is high despite awareness of adverse effects.

## INTRODUCTION

Primary dysmenorrhea is defined as "painful menses in women with normal pelvic anatomy, usually beginning during adolescence characterized by crampy pelvic pain shortly before or at the onset of menses lasting for 1 to 3 days".^1^ Painful uterine contractions and discomfort occur during these cycles mainly felt in lower abdomen but may radiate to back and the thigh.^2,3^ There are primary Dysmenorrhea without pathology and secondary to pelvic pathology.^4^

Use of medicines by the patient either on their own or on the advice of pharmacists without consulting registered health practitioners is self-medication.^5^ Due to easy accessibility, self-medication for primary dysmenorrhea is very common nowadays.^6^

Hence, this study is taken up to evaluate self-medication practice in primary dysmenorrhea among medical and nursing undergraduate students.

The main objective of this study is to find out the prevalence of self-medication among medical and nursing undergraduate students.

## METHODS

A descriptive cross-sectional study was done in medical and nursing undergraduate students of Kathmandu Medical College from November 2020 to March 2021. Ethical clearance (Ref: 2011202002) was obtained from the Institutional Review Committee of Kathmandu Medical College before the start of the study. Convenient sampling technique was used. The questionnaire did not contain any identifying details of the students and confidentiality was strictly maintained throughout the study. The participant's consent to participate in the study was implied when they clicked on the next button to answer the questionnaire and they had complete freedom either to decline or answer the questionnaire.

All medical and nursing under-graduate students willing to give consent were included in the study. Students having secondary dysmenorrhea (painful menstruation associated with underlying pathology) were excluded from the study.

Sample size was calculated as:

$$\begin{array}{ll} \text{n} & \text{= }{\text{Z}}^{\text{2}}\times \text{p}\times \text{q}/{\text{e}}^{\text{2}}\\ & \text{= }{\left(1.96\right)}^{\text{2}}\times 0.796\times 0.204/{(\text{0.05)}}^{\text{2}}\\ & \text{= }250 \end{array}$$

Where,

n = Sample size
z = 1.96 at 95% CI
p = prevalence of self-medication practice in primary dysmenorrhea among medical student (79.6%)^7^
q = 1-p
e = margin of error, 5%

The sample size is estimated to be 250 at 95% confidence interval. Adding 10% of non-response rate, the total sample size is 269.

The data was collected from online questionnaires delivered to participants via email, Viber, WhatsApp, messenger, etc because of COVID-19 pandemic. The data collection included demographic information, regularity of menstrual cycle, duration of menstrual discomfort, symptoms of primary dysmenorrhea, severity of pain assessment, self-medication used to relieve pain and awareness of self-medication. Pain assessment was done using a Visual Analogue Scale (VAS).

Responses from the Google form were downloaded in excel sheet format. Invalid data with invalid response was cleared and coding for responses was done. Data analysis was done in the Statistical Package of Social Sciences. Point estimate at 95% Confidence Interval was calculated along with frequency and proportion for binary data.

## RESULTS

**Figure 1:**
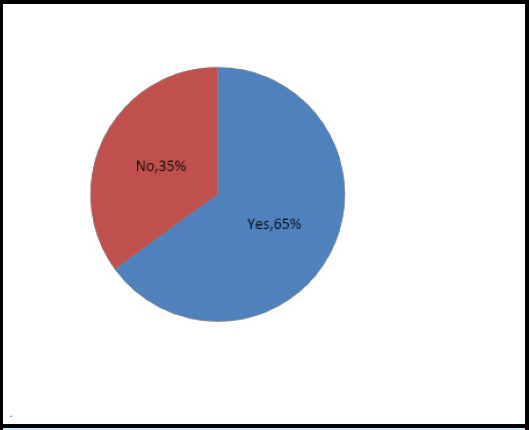
Prevalence of self medication

Out of 269 students, 175 (65%) have taken self-medication and only 20 (7.43%) visited registered healthcare practitioners which shows a high prevalence of self-medication among students (Figure 1).

Among various drugs used for self-medication, mefenamic acid tops the list with 121(48%) followed by ibuprofen 51 (20.3%) and paracetamol 41 (16.3%). Majority of them were aware of the negative impacts of self-medication 190 (70.6%) (Table 1).

**Table 1:** Prevalence of self-medication among students and most preferred medicines used in practice.

Variables	n (%)
**Self-medication among different stream**	
Medical	141 (80.57)
Nursing	34 (19.43)
**Preferred medicines**	
Mefenamic acid	121 (48)
Ibuprofen	51 (20.3)
Paracetamol	41 (16.0)
Mefenamic acid+dicyclomine	15 (6.0)
Diclofenac	12 (4.8)
Other	11 (4.3)
**Aware of negative impact of self-medication**	
Yes	190 (70.6)
No	79 (29.36)
**Outcome of self-medication**	
Cured	109 (62.28)
Improved	41 (23.42)
No improvement	25 (14.28)

Besides self-medication other self-care strategies of pain relief common among the students were 200 (74.43%) hot bath/bag, 234 (86.98%) rest, 114 (42.37%) reduced physical activities, 68 (25.27%) massage, 58 (21.56%) herbal remedy and 47 (17.47%) yoga.

Among 269 students, 192 (71.4%) were medical and 77 (28.6%) were nursing. Participants' age ranged between 17 to 28 years, and the mean age was 20.88±1.51 years (Table 2).

**Table 2:** Characteristics of menstrual cycle in participants with dysmenorrhea.

Variables	n (%)
**Regular menstrual cycle**	
Regular	195 (72.5)
Irregular	74 (27.5)
**Duration of Menstrual cycle**	
2-3 days	21 (71.8)
3-4 days	1 (0.4)
4-5 days	191 (71)
>5 days	56 (20.8)
**Amount of menstrual flow**	
Scanty	58 (21.6)
Moderate	190 (70.6)
Heavy	21 (7.8)
**Interval**	
<28 days	35 (13)
28-30	155 (57.6)
>30 days	79 (29.4)
**Menstrual discomfort**	
During menstrual period	43 (16)
During first 2 days	189 (70)
Throughout the cycle	37 (13)
**Symptoms**	
Nausea	83 (7.2)
Vomiting	27 (2.4)
Abdominal pain	182 (15.9)
Headache	73 (6.4)
Diarrhea	50 (4.4)
Back pain	187 (16.3)
Myalgia	133 (9.8)
Cramps	184 (16)
Loss of appetite	92 (8)
Irritability	157 (13.7)

The assessment of pain experienced by students according to different age groups and streams showed most of them experienced mild or moderate pain. The prevalence of mild or moderate pain was most common in the age group 21-25 years (Table 3).

**Table 3:** Severity of pain in dysmenorrhoea according to different age group and stream (n = 269)

Variables	Mild n (%)	moderate n (%)	Severe n (%)	Very severe n (%)	Worst n (%)
**Age**					
15-20	37 (32.6%)	33 (28.9%)	20(17.5%)	17 (14.9%)	7 (6.1%)
21-25	41 (27%)	45 (29.6%)	20(13.2%)	23 (15.1%)	23 (15.1%)
26-30	-	1 (33.3%)	-	2 (66.7%)	-
**Stream**					
Medical	43 (12.4%)	52 (27.1%)	33(17.2%)	34 (17.7%)	30 (15.6%)
Nursing	35 (45.5%)	27 (35.1%)	7(9.1%)	8 (10.4%)	-

## DISCUSSION

In the present study, the mean age of students was 20.88±1.51years which was more or less similar to Anand S et al.^8^ 195 (72.5%) participants had regular menstrual cycle which was similar to the study done in India by Jayanthi B et al.^9^ Among 269 participants who experienced dysmenorrhea, 191 (71%) of the participants reported 4-5 days of menstrual cycle and 189 (70%) reported pain for first 2 days of menstrual flow which was similar to Banikarim C et al. and Fatima A et al.^10,11^

Assessment of pain intensity and duration revealed that female undergraduates experience mild, moderate, severe, very severe and worst pain during menstruation which was similarly explained in the study done in China by Chen^12^ and Nigeria by Emmanuel et al.^13^

Most females in this survey experienced mild or moderate pain in the age group 21-25 years. The differences in the pain severity may be related to individual differences in pain perception and variability in pain threshold.

Majority of students 175 (65%) were taking self-medication, only very few of them visited registered healthcare practitioners 20 (7.43%) which shows a high prevalence of self-medication among students. Among various drugs used for self-medication, mefenamic acid tops the list with 121 (48%) which is similar to the study done by Marjoribanks J et al.^14^ and Feng X et al.^15^ mefenamic acid, being the widely used and preferred drug in primary dysmenorrhea. In contrast to our finding, study done by Farnandez et al.^16^ in Spain, most of the students used paracetamol as a common pain reliever. We also found that beside medicines students practised rest/relaxation, hot bath/ bag, herbal/home remedies, reduced physical activities and yoga for managing menstrual pain, similar to study of Anand S et al.^8^

Most of the students were aware of the negative impacts of self-medication 190 (70.6%) but only few opted for medical consultation. According to the students, reasons for not consulting were emergency use, prior experience in medicine, mild nature of illness, cost saving, lack of time to visit health care facilities and ease of access to non-prescribed medication. Most of the drugs were purchased from pharmacies (64.31%) rather than after visiting registered health care practitioners. One way to control this practice is by keeping check on buying and selling of unprescribed medicines with measures like educating pharmacists, formulating strict laws etc. Other important way is to educate students about self-medication and its consequences by lectures, seminars, and inclusion of topic in the curriculum itself. Alongside above steps, discussion on the topic among students, counselling by seniors or even faculty staff that not only make them aware but also encourage to bring change in behaviour also need to be exercised for tangible results.

## CONCLUSIONS

In primary dysmenorrhoea, self-medication practice among medical and nursing undergraduate students is excessive despite high awareness of adverse effects. So, along with strict laws and various awareness programmes beginning early at school college levels, measures to bring change in behavior should also be emphasised.
